# The Chaos of Combat: An Overview of Challenges in Military Mild Traumatic Brain Injury Research

**DOI:** 10.3389/fpsyt.2016.00085

**Published:** 2016-05-13

**Authors:** Nicholas D. Davenport

**Affiliations:** ^1^Research Service, Minneapolis VA Health Care System, Minneapolis, MN, USA; ^2^Department of Psychiatry, University of Minnesota, Minneapolis, MN, USA

**Keywords:** mTBI, concussion, military, neuroimaging

## Abstract

Mild traumatic brain injury (mTBI), or concussion, is among the most common injuries affecting Veterans of recent combat deployments. Military mTBI differs from civilian mTBI in fundamental ways that make assessment and diagnosis difficult, including a reliance on retrospective self-report and the potential influence of comorbid psychopathology. These unique features and their implications for research and clinical practice are summarized, and neuroimaging studies are discussed in the context of these complicating factors.

## Introduction

Mild traumatic brain injury (mTBI), or concussion, has been deemed the “signature injury” of Operation Enduring Freedom and Operation Iraqi Freedom (OEF/OIF) affecting 15–25% of American military service members deployed to these recent conflicts (1–3). Approximately 75% of these mTBI events have involved exposure to explosive blast (4, 5), which may have unique injury mechanisms (6, 7). Furthermore, even outside of deployments, mTBI is more common among military service members than civilians (3). Consequently, there has been substantial interest recently, both from clinical and scientific perspectives, in the identification of Service Members who have sustained mTBI to determine whether there are long-term effects. However, these efforts have been complicated by several unique features of military mTBI, such as reliance on retrospective self-report, overlap with symptoms of posttraumatic stress disorder (PTSD) and depression, and variability in assessment approaches. This manuscript presents an overview of some of these considerations, with brief discussions of their impact on clinical care and neuroimaging research.

## Mild Traumatic Brain Injury

According to the definition established by the American Congress of Rehabilitation Medicine (8), mTBI is characterized by a blow to the head (e.g., striking an object, being struck) accompanied by evidence of physiological disruption of brain function [i.e., loss of consciousness (LOC) less than 30 min, altered mental state (AMS), posttraumatic amnesia (PTA) less than 24 h, and neurological deficits]. While the major diagnostic symptoms of LOC, AMS, and PTA are generally limited in duration to the mTBI event itself, some physical (e.g., headaches and sleep disturbance), behavioral (e.g., irritability and disinhibition), and cognitive (e.g., difficulty in concentrating and memory problems) symptoms can persist for several days or weeks. These postconcussive symptoms (PCS) generally resolve gradually over time and, for the majority of individuals, are completely remitted within 3 months (9). However, a substantial subset of individuals report persistent PCS lasting several years or longer (10). Persistent PCS tend to be non-specific, correlate with anxiety and depression symptoms (11), and do not correspond well to objective neurological measures (12), leading to the hypothesis of a psychological rather than neurological basis. However, persistent PCS, PTSD, and depression are all risk factors for degenerative conditions, such as cerebral atrophy and chronic traumatic encephalopathy, raising the possibility that long-term effects of mTBI, including the relationship between PCS and psychopathology, are more complex (13).

## Diagnosis

Perhaps, the most striking differences between civilian and military mTBI are those inherent to the diagnostic process itself. Three elements in particular make the diagnosis of military mTBI unique: temporal proximity to the event, goals of establishing a diagnosis, and influence of trauma during and after the event (see Table 1). Approximately 60–75% of civilian mTBI events are reported to medical professionals shortly afterward (14, 15), creating a record proximal to the event. Diagnosis of mTBI, even in the hours and days following an event, involves clinical judgment and can be complicated by symptoms of acute stress disorder (16), delayed emergence of PCS, and individual differences in reporting; however, these factors are balanced by recency of memory and potential availability of witnesses. In contrast, documentation of military mTBI in theater has historically been rare and has only improved slightly since implementation of electronic medical records in 2010 (17). Therefore, diagnosis of military mTBI relies heavily on retrospective self-report of events that were traumatic in nature and involved states of altered consciousness and/or amnesia. When corroborating accounts are incorporated from independent witnesses, information is often still recounted through the individual being assessed (e.g., “My friend says I was unconscious for about 3 minutes”), limiting the independence of the source (18, 19). Lack of documentation at time of injury is largely due to reduced seeking of medical care for several reasons, including partial resolution of symptoms by the time access is possible (e.g., upon returning from patrol), fear of unnecessary reduction in duty, and reduced awareness of acute symptoms in the context of stressful events. In addition to changing the nature of the diagnostic process, it is possible that differences in acute care (e.g., rest) have effects on recovery, neurobiological processes, and long-term outcomes.

**Table 1:** **Summary of key points of comparison between civilian and military mTBI**.

	Civilian mTBI	Military mTBI
Typical time since injury at diagnosis	Hours to days	Months to years
Goals of diagnosis^a^	Documentation of injury, prospective monitoring of course, direct acute care to limit progression and speed recovery	Documentation of injury, identify potential PCS, identify those at risk for progressive neurodegeneration
Involvement of trauma	Common, typically limited to event, may affect reporting	Common, often extends beyond mTBI event (e.g., securing self, others, and equipment following explosion), may confound with experience and reporting of mTBI symptoms (e.g., difficult to differentiate altered consciousness from confusion of threatening situation)

*^a^At typical time of diagnosis (i.e., hours to days for civilian mTBI, months to years for military mTBI)*.

While there are obvious limitations to relying on retrospective accounts of events that occurred months or years previously, there are also more subtle consequences. The primarily retrospective approach to diagnosis will naturally overestimate the prevalence of persistent PCS relative to a prospective approach due to selection bias. To improve identification of veterans who experienced deployment-related mTBI, the Veterans Health Administration (VHA) developed a TBI clinical reminder screening questionnaire to be administered to all OEF/OIF Veterans seeking care at a VHA medical facility (20), with positive screens referred to specialists for formal mTBI assessment. While the merits and effectiveness of this strategy have been debated in depth elsewhere [e.g., Ref. (21)], it has highlighted the question of what the goal of retrospective diagnosis should be. In the acute phase of mTBI, diagnostic assessment of symptoms can be used to determine severity and make judgments about reductions in activities, return to work or play, and monitoring of course. In this sense, early diagnosis has the goal of limiting underlying neurodegenerative processes and speeding recovery, and following full recovery the diagnosis is typically considered historical rather than ongoing. Retrospective diagnoses conducted months or years later cannot achieve the goal of limiting damage, and the value of an historical mTBI diagnosis has not been established. While it may be beneficial to identify veterans who are experiencing persistent PCS or who may be at risk for progressive neurodegeneration (13), the discrepancy between the two diagnostic approaches limits the generalization of scientific knowledge and clinical practice between civilian and military contexts.

Finally, military mTBI is distinct from civilian mTBI in terms of context, specifically, the prominence of psychological trauma. While civilian mTBI often involves highly stressful or potentially life-threatening contexts (e.g., motor vehicle accidents and assaults), the need for immediate action (i.e., during acute symptoms) to remove oneself from further harm is rare. In contrast, military mTBI events, 75% of which involve explosive blasts (e.g., mortars and roadside bombs), often require the person to respond quickly by removing themselves or others from harm, assess damage or casualties, secure equipment, identify additional threats, and respond according to their military operational specialty (22). Consequently, it can be difficult for a clinician to differentiate confusion due to altered consciousness from confusion inherent to an ambiguous threatening situation, especially when based on retrospective report (17, 19). Furthermore, due to immediate need for action, often relying on reflexive heavily trained behaviors, the individual may not be aware of acute symptoms until much later or may attribute these symptoms to the stressful situation rather than biological insults, further contributing to low rates of mTBI reporting proximal to the event.

Conversely, a high rate of comorbidity between mTBI and PTSD has been consistently reported among recent veterans. In an early and prominent example of this relationship, Hoge et al. (1) demonstrated that 43.9% of the OIF Soldiers who reported mTBI with LOC met criteria for PTSD, compared to 27.3% of those reporting mTBI without LOC, 16.2% of those reporting non-mTBI injuries, and 9.1% of uninjured Soldiers. It has also been consistently reported that the association between mTBI and higher levels of PCS is no longer significant after accounting for PTSD and/or depression symptoms (1, 11, 23, 24), suggesting a psychological rather than physiological basis for persistent PCS. In addition to making the distinction of PTSD from chronic effects of mTBI even more difficult, this relationship raises the possibility that the experience of PTSD affects diagnosis of mTBI either directly or indirectly, such as by misattribution of symptoms by the clinician or the patient. Two recent independent reports investigating inconsistency of reported mTBI experiences between the end of a deployment and 6–9 months later both determined that higher PTSD symptom levels correspond to increased likelihood of inconsistent mTBI reporting, specifically changing from no initial report of mTBI to later endorsement of such experiences (25, 26). Taken together with the typical retrospective nature of diagnosis and high rate of comorbidity with PTSD, the demonstration that mTBI reporting changes over time raises concerns about reliability of military mTBI diagnosis.

## Loss of Consciousness and Blast Exposure

In response to the challenges posed by military mTBI diagnosis, particularly the reliability of retrospective self-report and influence of psychopathology, there has been interest in determining whether specific elements of the mTBI event uniquely relate to outcomes and underlying neuropathology. Exposure to explosive blast has received substantial attention in this regard due to its specificity to military combat and evidence from animal models demonstrating unique biophysical damage mechanisms relative to traditional acceleration–deceleration forces involved in civilian injuries (4, 6, 7). Blast exposure is particularly common among OEF/OIF veterans due to the prominence of improvised explosive devices, combined with advanced body protection improving survival rates (3, 22). While blast exposure is associated with increased rates of PTSD (3), especially the re-experiencing symptom domain (27), it is also associated with higher rates of combat exposure (28, 29), but not with more persistent PCS (5), suggesting that blast exposure may be psychologically rather than physiologically traumatic.

Loss of consciousness is another logical candidate as a unique element of mTBI, because it is more objective than AMS, often observed by others, and may be indicative of somewhat greater magnitude of injury. In a pair of studies comparing independent effects of mTBI and LOC on psychological measures, Verfaellie et al. (24, 30) demonstrated a specific association between LOC and emotional PCS, but no relationship of mTBI or LOC with neuropsychological performance; however, neuropsychological performance was associated with anxiety and depression symptoms. Notably, corroborative report was used to make 25 out of the 28 determinations of LOC but was absent in all cases of AMS (18). While this serves to highlight the value of witness report to the assessment of LOC, it also highlights the possibility that the inclusion of third-party report in mTBI assessment may confound the determination of loss vs. alteration of consciousness with overall diagnostic confidence.

## Neuroimaging

Neuroimaging has been used to further characterize the neural underpinnings of mTBI and potential effects of PTSD, LOC, and blast exposure. Based on evidence of diffuse axonal injury associated with moderate and severe TBI (31, 32), diffusion tensor imaging (DTI) and resting state functional magnetic resonance imaging (rsfMRI) have been used extensively to assess structural and functional connectivity, respectively. Briefly, DTI models water diffusivity within each voxel as a tensor of three orthogonal vectors with varying magnitude (33). Because diffusion in white matter is directionally restricted by axon walls, myelin, and other cytoskeletal elements, the two primary DTI measures used to describe white matter integrity are the degree to which diffusion has a single primary orientation [fractional anisotropy (FA)] and the total magnitude of diffusion across the three vectors [mean diffusivity (MD)] (34). In general, healthy white matter is characterized by high FA and low MD (35), and studies of civilian mTBI have typically reported lower FA and higher MD, relative to uninjured controls, in the chronic phase (36–39). Studies of veterans have been more variable, with early studies reporting no differences in FA or MD at the level of individual regions or voxels within deployed veterans (40, 41), although one group reported reduced anisotropy among Service Members medically evacuated for combat injuries including mTBI compared to those whose injuries did not include mTBI (42). Notably, diagnoses of mTBI in the two former studies were made several years after the injury, whereas the latter positive result was based on diagnoses made within 90 days of the mTBI event, though this may reflect recency of injury rather than proximity of the report to the injury. While PTSD symptoms or diagnoses were not explicitly considered in these early reports, subsequent studies that included PTSD symptom severity as a regressor (43) or excluded participants meeting diagnostic criteria for PTSD at the time of study (44) found evidence of lower FA associated with mTBI in widespread white matter. When looking specifically at effects of PTSD within a military mTBI sample, one study reported a negative correlation between FA and PTSD symptom severity (45), whereas another reported higher anisotropy and lower MD associated with PTSD diagnosis (46). A follow-up to the latter demonstrated that both mTBI and PTSD were associated with persistent PCS, but mTBI was associated with fewer regions of abnormal MD while PTSD was associated with more such regions (23). Overall, this pattern of decreased FA across studies suggests that white matter integrity is indeed lower among veterans reporting a history of mTBI, though the association appears to be weaker than in civilian studies, possibly due to the influence of PTSD. While lower FA may represent white matter damage as a consequence of mTBI, additional longitudinal studies are required to establish this interpretation.

Because DTI uses a simple tensor model, it can only account for one fiber orientation per voxel and underestimates anisotropy in voxels containing crossing or diverging fibers. Using a measure of generalized FA (GFA) that accounts for multiple fiber orientations, we previously reported that GFA was more sensitive than FA to effects of PTSD, though neither measure demonstrated an effect of mTBI (46). This improved sensitivity of GFA suggests that crossing and diverging fibers, rather than well-organized tracts, may be particularly involved in PTSD pathology. As advanced measures (e.g., GFA) and techniques (e.g., diffusional kurtosis imaging) become more common, the underlying pathology will likely be clarified further.

Several studies have reported an association between LOC and lower FA, relative to AMS, in widespread white matter regions (18, 47, 48). Similarly, Sorg et al. (49) found that while there was no overall effect of mTBI on FA, a subset of the mTBI group who also demonstrated executive function deficits had lower FA in widespread frontal and subcortical white matter. Furthermore, participants with LOC were more likely to demonstrate executive function deficits and had reduced FA in ventromedial prefrontal white matter, demonstrating a relationship among LOC, executive function, and white matter integrity. This is consistent with reports that LOC is associated with PCS and verbal memory performance through its relationship with white matter integrity even after accounting for PTSD symptoms (18, 48). Finally, one study reported a negative relationship, observed only among veterans with LOC, between a measure of blast load and FA in two subcortical white matter regions, demonstrating an additional dose–response relationship within the more affected group (18). Overall, these results indicate that mTBI involving LOC demonstrates unique relationships with white matter integrity, cognitive function, and symptom expression. Future work to characterize these relationships further, especially their stability over time, has substantial potential to advance understanding of effects of mTBI.

While there have been relatively fewer studies of functional connectivity in military mTBI, two, in particular, have revealed novel features. First, Costanzo et al. (50) reported that left anterior default mode network connectivity (i.e., correlated activity between medial frontal and posterior cingulate cortex) was positively associated with FA of the left cingulum (i.e., the structural pathway between the two functional seeds) and negatively associated with PTSD re-experiencing symptoms, indicating PTSD-related functional disconnection. These associations were observed among veterans with mTBI, but not among veterans without mTBI, suggesting that they represent an emergent property of mTBI neuropathology rather than naturally occurring intersubject variability. Second, Robinson et al. (29) tested a wide range of contrasts involving blast exposure, mTBI symptoms, and injury mechanism (i.e., blast vs. impact) to determine which grouping variable best discriminated cingulate connectivity. Based on comparisons of these tests, the most critical factor was determined to be exposure to a blast at a distance of less than 10 m, regardless of acute mTBI symptoms. Specifically, these results indicate that the presence of the injury mechanism (i.e., close-range blast) may be more critical to understanding the associated neuropathology than the presence of symptoms that are, at least in part, subjective and variable across individuals. These studies demonstrate that functional connectivity may provide valuable information, complementary to that from structural connectivity studies, for the characterization of neuropathology underlying military mTBI.

## Conclusion

While there are clear challenges inherent to establishing diagnoses based primarily on retrospective self-report of events that, by definition, involve states of altered consciousness, there are also substantial opportunities to better understand the dynamic relationships among psychological and physiological trauma. Innovative study designs that parse individual elements of the mTBI event, subsequent experiences and symptoms, and underlying traits (e.g., personality, genetic predisposition, and premorbid psychopathology) will be valuable and likely necessary.

## Author Contributions

The author confirms being the sole contributor of this work and approved it for publication.

## Conflict of Interest Statement

The author declares that the research was conducted in the absence of any commercial or financial relationships that could be construed as a potential conflict of interest.
